# New insights in ventriculo-arterial coupling and ventricular shape in repaired tetralogy of Fallot: a retrospective cohort study

**DOI:** 10.1186/1532-429X-18-S1-O118

**Published:** 2016-01-27

**Authors:** Giovanni Biglino, Nikesh Arya, Kristin McLeod, Silvia Schievano, Andrew Taylor

**Affiliations:** 1 grid.83440.3b0000000121901201Institute of Cardiovascular Science, University College London, London, UK; 2 grid.420468.cGreat Ormond Street Hospital for Children, London, UK; 3 Simula Lab, Oslo, Norway

## Background

Severe pulmonary regurgitation (PR%) and consequent right ventricular (RV) enlargement are observed features in repaired tetralogy of Fallot (ToF). Due to ventricular interactions, the shape and function of the left ventricle (LV) can also be affected. It has been shown, for instance, that ToF patients exhibit reduced longitudinal strain as a possible sign of LV dysfunction, despite normal ejection fraction (EF). Literature has also indicated that LV dysfunction in ToF can have prognostic value. This study thus sought to investigate ventriculo-arterial coupling on the systemic side and LV shape by means of CMR analysis.

## Methods

A retrospective cohort of ToF patients (n = 201) was analysed. Patients with late repair (>1 year) and BT/Waterston shunts were excluded. All patients had CMR examination. The phase contrast flow sequences in aortic position were analysed to derive aortic wave speed and wave intensity, the latter defined as the product of velocity differentials with fractional changes in aortic area and taken as an indicator of the working efficiency of the LV and ventriculo-arterial coupling. Wave intensity data were stratified according to PR% and analysed accordingly. Additionally, multivariable regression analysis was performed on the whole cohort, assessing the effect of PR%, aortic distensibility, sex and age on wave intensity magnitude. Furthermore, short axis stack sequences of the RV and LV were used as inputs for statistical shape analysis, building a statistical shape atlas for both RV and LV for different PR% (i.e. none, mild, moderate, severe).

## Results

Functional CMR data revealed enlarged RV (p < 0.001) with severe PR%, as expected, while LV volume did not change significantly across the strata. LVEF was significantly lower for severe PR% (61 ± 6 vs. 67 ± 7, p = 0.002), albeit still in the normal range. Wave intensity magnitude was significantly (p = 0.01) lower in the presence of PR%, indicating less favourable aortic ventriculo-arterial coupling; however it did not progressively decrease with increasing PR%. Patients with increasing PR% severity did exhibit stiffer aortas (p = 0.01) according to wave speed results and a multivariable regression model showed that aortic distensibility was in fact the only significant (p = 0.001) predictor of wave intensity magnitude, particularly of the forward compression wave, typically considered as a surrogate for ventricular contractility. Statistical shape atlases were also successfully calculated, providing additional insight into RV and LV shapes.

## Conclusions

Wave intensity analysis and statistical shape analysis confirm changes in RV and LV function and shape in patients with repaired ToF. Patients with some degree of PR%, even mild, exhibited lower forward compression wave in the aortic wave intensity profile, suggesting unfavourable ventriculo-arterial coupling, but analysis revealed that reduced aortic distensibility is the main predictor for changes in LV efficiency.

